# Correlation of bilateral M1 hand area excitability and overall functional recovery after spinal cord injury: protocol for a prospective cohort study

**DOI:** 10.1186/s12883-024-03705-0

**Published:** 2024-06-22

**Authors:** Chunqiu Dai, Xiaodong Lin, Baijie Xue, Xiao Xi, Ming Gao, Xinyu Liu, Tao Han, Qiaozhen Li, Hua Yuan, Xiaolong Sun

**Affiliations:** 1 Department of Rehabilitation Medicine, Xijing Hospital, Air Force Medical University (Fourth Military Medical University), Xi’an, 710032 PR China; 2 Lintong Rehabilitation and Convalescent Centre, Xi’an, 710600 PR China

**Keywords:** Spinal cord injuries, Cortical excitability, Motor evoked potential, Activities of daily living, Motor cortex

## Abstract

**Background:**

After spinal cord injury (SCI), a large number of survivors suffer from severe motor dysfunction (MD). Although the injury site is in the spinal cord, excitability significantly decreases in the primary motor cortex (M1), especially in the lower extremity (LE) area. Unfortunately, M1 LE area-targeted repetitive transcranial magnetic stimulation (rTMS) has not achieved significant motor improvement in individuals with SCI. A recent study reported that the M1 hand area in individuals with SCl contains a compositional code (the movement-coding component of neural activity) that links matching movements from the upper extremities (UE) and the LE. However, the correlation between bilateral M1 hand area excitability and overall functional recovery is unknown.

**Objective:**

To clarify the changes in the excitability of the bilateral M1 hand area after SCI and its correlation with motor recovery, we aim to specify the therapeutic parameters of rTMS for SCI motor rehabilitation.

**Methods:**

This study is a 12-month prospective cohort study. The neurophysiological and overall functional status of the participants will be assessed. The primary outcomes included single-pulse and paired-pulse TMS. The second outcome included functional near-infrared spectroscopy (fNIRS) measurements. Overall functional status included total motor score, modified Ashworth scale score, ASIA Impairment Scale grade, spinal cord independence measure and modified Barthel index. The data will be recorded for individuals with SCI at disease durations of 1 month, 2 months, 4 months, 6 months and 12 months. The matched healthy controls will be measured during the same period of time after recruitment.

**Discussion:**

The present study is the first to analyze the role of bilateral M1 hand area excitability changes in the evaluation and prediction of overall functional recovery (including motor function and activities of daily living) after SCI, which will further expand the traditional theory of the predominant role of M1, optimize the current rTMS treatment, and explore the brain-computer interface design for individuals with SCI.

**Trial registration number:**

ChiCTR2300068831.

**Supplementary Information:**

The online version contains supplementary material available at 10.1186/s12883-024-03705-0.

## Introduction

Spinal cord injury (SCI) often occurs in young adults [1]. Although the injury site is in the spinal cord, significantly decreased excitability in the primary motor cortex (M1), especially in the lower extremity (LE) area, has been found in the early stage of SCI [2, 3]. Therefore, targeted regulation of the M1 LE might promote motor recovery. Unfortunately, some studies indicate that even after four weeks of continuous M1 LE area repetitive transcranial magnetic stimulation (rTMS), the gait function of individuals with SCI did not improve significantly compared with that of individuals in the sham group [4, 5]. One possible reason for this may be due to the deep location of the M1 LE area, the small size, and the limited efficacy of the current figure-8, circular rTMS coils [6]. The H-coil is designed to achieve effective stimulation of deep neuronal regions. However, the H-coil is disadvantageous because of its wider electrical field distribution and lower stimulation accuracy in the brain [7]. Therefore, the latest rTMS guidelines do not recommend treating motor dysfunction (MD) after SCI [8]. Identifying new targets of rTMS is a high priority.

Using multiunit recordings, one study reported that the hand knob area of the precentral gyrus (the location of the M1 area) of an individual with SCI is tuned to the entire body. Interestingly, this area contains a compositional code that links matching movements from upper extremities (UE) and LE [9]. The compositional code is the movement-coding component of neural activity and is then transferred to another limb by changing only the limb-coding component. All four limbs movement-coding components could be measured in the M1 hand area. Therefore, the M1 hand area might contain a compositional code that links all four limbs together. Similarly, through precision functional magnetic resonance imaging (fMRI), Gordon EM et al. demonstrated that the classic M1 area is functionally interrupted by regions with distinct connectivity and alternating with effector-specific (foot, hand and mouth) areas [10]. Very recently, Lorach H et al. attempted to restore communication between the brain and spinal cord with a brain–spine interface (BSI) that enabled chronic tetraplegia individuals with SCI to stand and walk naturally. Interestingly, the region that responded more robustly to the intention to move the LE identified by electrocorticography (ECoG) signals basically covered the M1 hand area [11]. According to the classic M1 organization as a continuous homunculus from toe to head, the hand area is shallower and larger than the LE area [12]. Therefore, compared with M1 LE motor-evoked potentials (MEPs), M1 hand area MEPs are more commonly used because of the high detection rate of MEP amplitude in the clinic. Taken together, these findings clarify the changes in the excitability of the bilateral M1 hand area after SCI and its correlation with motor recovery and the ability to perform activities of daily living (ADL). This is of paramount significance for specifying the therapeutic parameters of rTMS for SCI motor rehabilitation.

In our recent study, we explored the correlation between corticospinal excitability (CSE, reflected by the MEP) changes in the bilateral M1 hand area after SCI and overall functional recovery through a cross-sectional study [13]. The results showed that the CSE of the dominant hemisphere (DH) M1 hand area was significantly greater than that of the nondominant hemisphere (NDH) M1 hand area in healthy controls, whereas in individuals with SCI, the opposite phenomenon occurred: patients exhibited a decrease in the CSE of the DH M1 hand area, while the M1 hand area MEP hemispheric CSE conversion was correlated with ADL ability and LE motor function in individuals with SCI. The closer the degree of M1 hand area MEP hemispheric conversion was to that of healthy controls, the better the extremity motor function/ADL ability patients achieved. To verify the phenomenon found in our previous cross-sectional study, we designed this cohort study to confirm the relationship between hemispheric M1 hand area excitability conversion and overall functional recovery with the extension of the course of SCI up to one year through multilevel brain measurements consisting of MEPs for the CSE, paired-pulse transcranial magnetic stimulation (ppTMS) for intracortical excitability and functional near-infrared spectroscopy (fNIRS) for brain network activation.

## Methods

### Study design and setting

The present study is a 12-month prospective cohort study that does not involve intervention and will be carried out between September 2023 and December 2026 at Xijing Hospital, a tertiary academic medical care institution with 3200 beds in Xi’an, China. Neurophysiological and overall functional status data will be collected for these participants. The outcomes will be assessed in healthy controls and individuals with SCI at disease durations of 1 month, 2 months, 4 months, 6 months and 12 months. The Standard Protocol Items: Recommendations for Interventional Trials (SPIRIT) statement 2013 [14] was used as the reporting guideline for this protocol.

### Sample size calculation

The sample size calculation was conducted via PASS 11.0 software based on the results of the MEP in our cross-sectional study [13], which showed that approximately 27% of healthy controls had a greater M1 hand area CSE in the NDH (P2 = 0.26), and approximately 70% of individuals with SCI had a greater M1 hand area CSE in the NDH (P1 = 0.7, R1 = P1/P2). Other parameters were set as follows: a significance level of α = 0.05 (two tails), power (1–β) = 90%, and standard deviation = 1. Therefore, a sample size of 25 individuals with SCI and 25 healthy controls was used. Considering the dropout rate in the cohort study, as the number of subjects increased by 20%, a minimum of 60 participants (30 individuals with SCI and 30 healthy controls) were needed.

### Participants

#### Inclusion and exclusion criteria

The inclusion criteria for individuals with SCI were as follows: (a) 18–75 years old, right-hand dominant (Edinburgh Handedness Inventory); (b) diagnosed with SCI [15]; (c) stable MEP in the M1 hand area was detectable; (d) no cognitive impairment (Mini-Mental State Examination score ≥ 24) [16]; (e) disease course ≤ 1 month (from SCI onset to first assessment); and (f) agreed to participate in this study and signed the informed consent form. Exclusion criteria: (a) serious nervous system disease, unable to tolerate relevant tests; (b) upper limb fracture, arteriovenous fistula or other diseases leading to movement disorder; (c) ferromagnetic implants in the head or neck; (d) implantation of a cardiac pacemaker; (e) severe coagulation disorder, severe cardiac insufficiency or uncontrollable hypertension: systolic blood pressure > 160 mmHg or diastolic blood pressure > 100 mmHg; (f) other serious systemic diseases such as tumors; (g) taking drugs that may affect the TMS examination within a week, including baclofen, diazepam and other drugs [17]; (h) absolute contraindications for TMS examination.

The inclusion criteria for healthy controls were as follows: (a) 18–75 years old, right-hand dominant (Edinburgh Handedness Inventory); and (b) stable MEPs. Exclusion criteria included the above situations in which TMS could not be performed.

#### Recruitment and sample selection

Potential participants will first be screened by nurses based on inclusion and exclusion criteria and informed about the study during a consultation with nurses if they meet the inclusion criteria. To assess the external validity of the recruited sample of participants, age and gender will be collected for those participants who refuse to participate in the study [18]. If potential participants are interested in participating in this study, their names will be placed on a trial list. The investigator will contact the participants and explain the procedure. If the participants agree, an informed consent form will be sent. If the participants have any questions, they can contact the investigator.

Upon receipt of the signed informed consent form, the participants will be registered in the electronic case report form (eCRF), and the basic information will be recorded at baseline. Then, the doctor will assess the neuroelectrophysiology data, and the ability to perform ADLs and motor function will be evaluated by an occupational therapist. The participants received a reminder or were contacted by phone at subsequent testing time points. Measurements are recorded by the nurse specialist in the Electronic Patient Record.

After recruitment, individuals with SCI will be measured at disease durations of 1 month, 2 months, 4 months, 6 months and 12 months, including single pulse TMS (spTMS) to evaluate the CSE in bilateral M1 hand areas, ppTMS to evaluate the intracortical excitability in bilateral M1 hand areas, and fNIRS to evaluate bilateral M1 hand area blood flow. All subjects will receive an assessment scale. The matched healthy controls will be measured during the same period after recruitment (flow chart, Table 1).

**Table 1:** SPIRIT-figure of the measurement instruments and time of assessment

		Study period						
		Enrolment						
Timepoint	Type	Healthy control	1 month (SCI-T1)		2 months (Control/SCI-T2)	4 months (Control/SCI-T3)	6 months (Control/SCI-T4)	12 months (Control/SCI-T5)
**Enrolment**								
Eligibility screen		**×**	**×**					
Informed consent		**×**	**×**					
**Assessments**								
spTMS (RMT, MEP)	Neuroexcitability	**×**	**×**		**×**	**×**	**×**	**×**
ppTMS (SICI, ICF, LICI)	Neuroexcitability	**×**	**×**		**×**	**×**	**×**	**×**
fNIRS	Neuroexcitability	**×**	**×**		**×**	**×**	**×**	**×**
**Prognostic evaluation**								
Motor Score	Motor Function	**×**	**×**		**×**	**×**	**×**	**×**
MAS	Motor Function	**×**	**×**		**×**	**×**	**×**	**×**
AIS Grade	Injury Extent	**×**	**×**		**×**	**×**	**×**	**×**
SCIM	ADL ability	**×**	**×**		**×**	**×**	**×**	**×**
MBI	ADL ability	**×**	**×**		**×**	**×**	**×**	**×**
**Basic characteristic**								
Age	Basic Characteristic	**×**	**×**					
Gender	Basic Characteristic	**×**	**×**					
Height, Weight	Basic Characteristic	**×**	**×**					
Education	Basic Characteristic	**×**	**×**					

*Abbreviations* SCI: spinal cord injury; spTMS: single pulse TMS; ppTMS: paired-pulse TMS; fNIRS: functional near-infrared spectroscopy; RMT: resting motor threshold; MEP: motor evoked potentials; SICI: short-interval intracortical inhibition; ICF: intracortical facilitation; LICI: long-interval intracortical inhibition; AIS: American Spinal Injury Association (ASIA) impairment scale; AIS: MAS: modified Ashworth scale; MBI: modified Barthel index; SCIM: spinal cord independence measure

**Flow chart:**
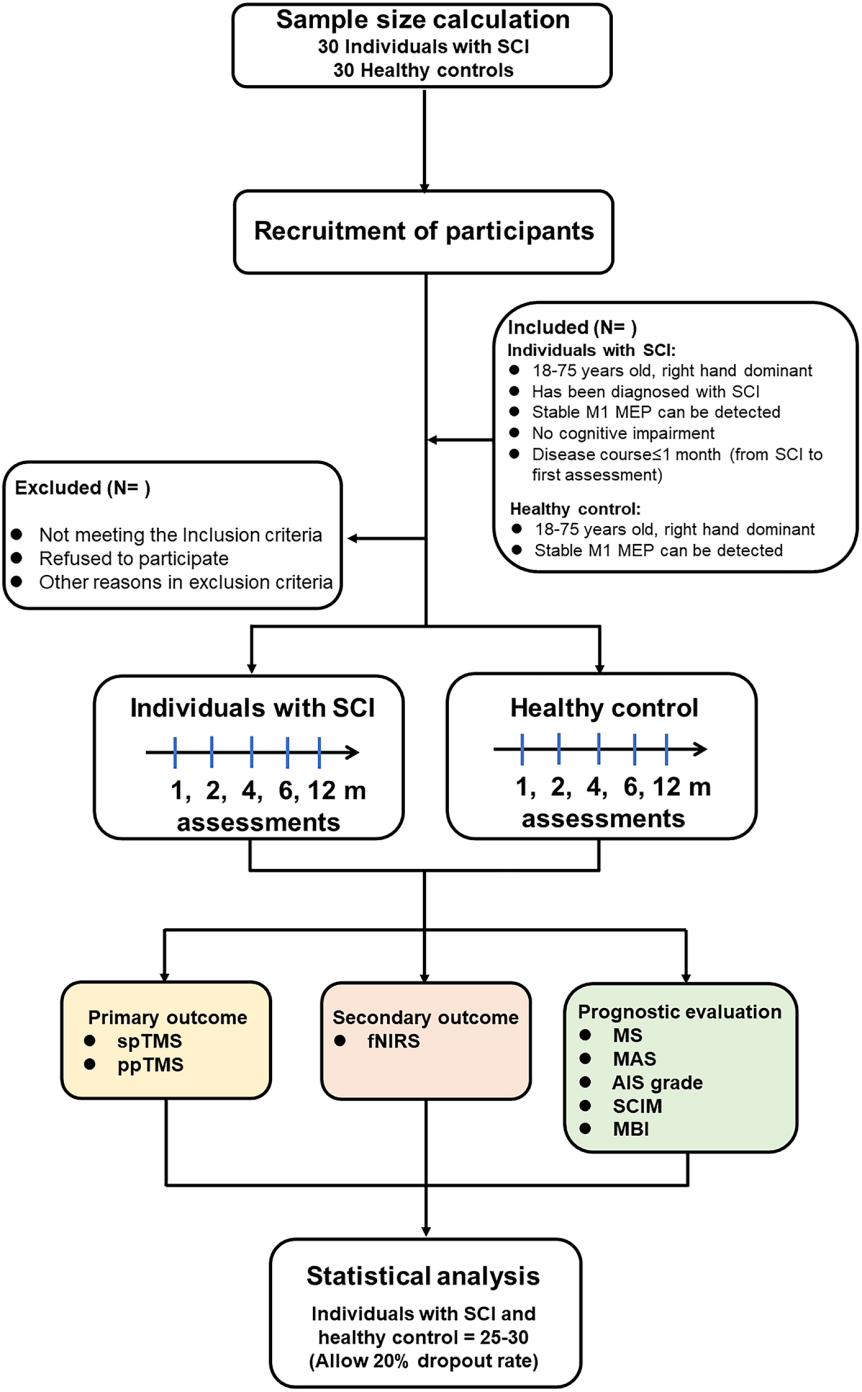
Flow diagram of the study design. *Abbreviations* SCI: spinal cord injury; spTMS: single pulse TMS; ppTMS: paired-pulse TMS; fNIRS: functional near-infrared spectroscopy; MS: motor score; AIS: American Spinal Injury Association (ASIA) impairment scale; MAS: modified Ashworth scale; MBI: modified Barthel index; SCIM: spinal cord independence measure

Every participant data file will be given a number and stored in a secure and accessible place. The files will be kept for three years after completion of the study.

### Outcome measures

#### Primary outcome

##### spTMS

To evaluate the changes in the M1 hand area CSE between individuals with SCI and healthy controls, the amplitude of the MEP and the resting motor threshold (RMT) will be detected through spTMS equipment (MEP-9404 C, Japan).

**Fig. 1:**
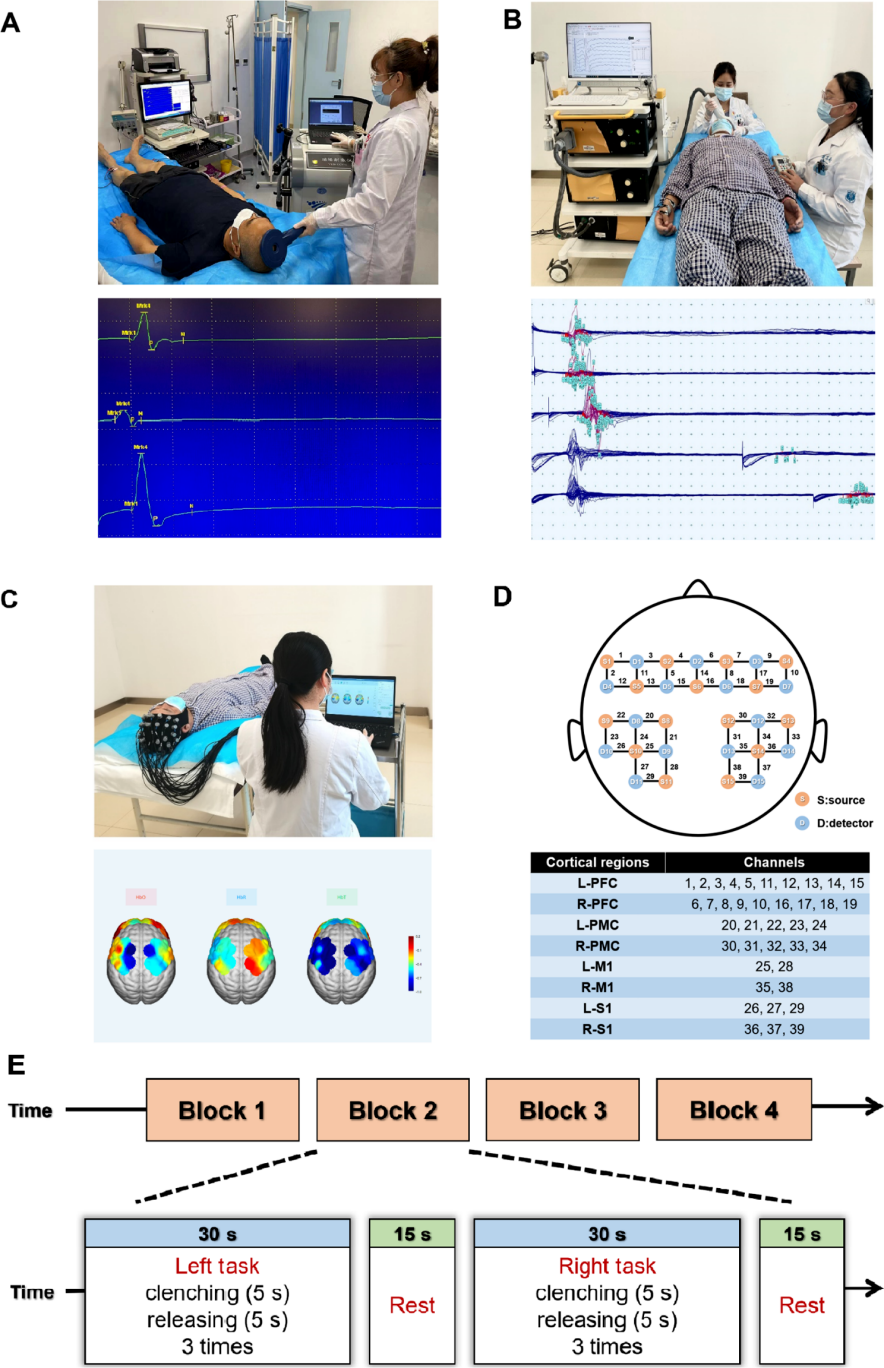
Bilateral M1 hand area excitability measurement and fNIRS measurement. **A**. spTMS; **B**. ppTMS; **C**. fNIRS measurement; **D**. fNIRS channel layout and ROI; **E**. Handgrip task design. During the test, the subjects were required to perform the grip experiment, which included the following steps: 1) left-hand clenching for 5 seconds, releasing for 5 seconds, and repeated three times; 2) 15 s resting state; and 3) right-hand clenching for 5 seconds, releasing for 5 seconds, and repeated three times. *Abbreviations* spTMS: single pulse TMS; ppTMS: paired-pulse TMS; fNIRS: functional near-infrared spectroscopy

The MEP of the M1 hand area was recorded by the magnetic stimulation coil placed in the M1 hand area on the left side of the skull. The recording surface Ag-AgCl electrode and reference electrode were pasted on the abdomen of the abductor pollicis brevis muscle of the right hand and connected to the recording equipment. A single-pulse TMS is delivered to the hotspot under 80% of the maximum stimulator output. The MEP latency and amplitude of the abductor pollicis brevis muscle were recorded. RMT is defined as the minimal amount of stimulus intensity producing MEP peak-to-peak ≥ 50 µV in at least 5 out of 10 consecutive tracks [19]. The protocol used for the right M1 hand area was the same as that described above (Fig. 1A).

##### ppTMS

To evaluate the changes in intracortical excitability of the M1 hand area between individuals with SCI and healthy controls, the amplitude of ppTMS (Neurosoft, Russia) will be measured.

During the measurements, TMS was applied twice over the M1 area. The first pulse is defined as the conditional stimulus, and the second pulse is defined as the test stimulus. By determining the change in the test stimulus relative to the baseline MEP, the excitability or inhibitory state of the M1 hand area can be noninvasively measured. The inhibitory indicators included short-interval intracortical inhibition (SICI, pulse interval 2.5 ms) and long-interval intracortical inhibition (LICI, pulse interval 100 ms). The commonly used excitability indicator is intracortical facilitation (ICF, pulse interval 12 ms). Previous studies have confirmed that ICF is related to N-methyl-D-aspartic acid (NMDA) receptor function, while SICI and LICI are related to gamma-aminobutyric acid A (GABA_A_) and gamma-aminobutyric acid B (GABA_B_) receptor function, respectively [20] (Fig. 1B).

#### Secondary outcomes

Based on the theory of neurovascular coupling (NVC), which states that increased neural activity can drive changes in local blood flow through neurovascular coupling [21], we utilized fNIRS (NirSmart, HuiChuang, China) to evaluate changes in blood flow in the M1 area between individuals with SCI and healthy controls.

The fNIRS test method parameters have been previously described [19]. The near-infrared electrode cap can detect near-infrared light absorption at 780, 805 and 830 nm, and the data can be converted into oxygenated hemoglobin (HbO), deoxyhemoglobin (HbR) and total hemoglobin (HbT) values. The detection channels were located according to Montreal Neurological Institute (MNI) coordinates, and the regions of interest (ROIs) were the bilateral M1, primary sensory cortex (S1), premotor cortex (PMC) and prefrontal cortex (PFC) (Fig. 1C D). During the test, the subjects were required to perform the grip experiment [19], which included (1) clenching the left hand for 5 s, releasing for 5 s, and repeating the process three times; (2) resting for 15 s; and (3) clenching the right hand for 5 s, releasing for 5 s, and repeating the process three times (Fig. 1E).

### Overall functional recovery evaluation

To evaluate the overall functional recovery of individuals with SCI, we utilized different scales and assessments for motor function and ADL ability evaluation.

#### Motor score (MS)

The MS mainly evaluates the strength of key muscles innervated by different spinal nerves. The total score is 100, consisting of the upper extremity MS (UEMS) and lower extremity MS (LEMS) (Supplemental Table 1).

#### Modified ashworth scale (MAS)

The MAS is the most commonly used clinical scale for measuring muscle tone [22]. The classification standards included 0, 1, 1+, 2, 3, and 4. (Supplemental Table 2).

#### ASIA impairment scale (AIS) grade

The AIS grade [23] mainly determines the degree of SCI (complete/incomplete injury) and can be divided into five scales: A, B, C, D and E. A indicates complete injury, B-D indicates incomplete injury, the degree of injury decreases successively, and E indicates normal sensorimotor function (Supplemental Table 3).

#### Spinal cord independence measure (SCIM)

The SCIM mainly evaluates the prognosis and overall quality of life of individuals with SCI [24]. The total score is 100. There are 17 items in total, of which Item 12 (**SCIM12**) is of particularly important for the evaluation of motor function in individuals with SCI [25]. The total SCIM12 is 8 (Supplemental Table 4).

#### Modified barthel index (MBI)

The MBI [26] mainly evaluates the independent living ability of individuals with SCI and includes 10 items. Compared with SCIM, the MBI focuses on patients’ self-care ability in daily life, which indicates whether patients can operate independently when completing the above actions. The total score is 100 (Supplemental Table 5).

### Data management

All recorded data will be collected in the CRF paper form. One authorized clinical staff member will enter the data into the eCRF. The entered data will be subjected to plausibility, monitoring and medical review. Implausible or missing data will be queried.

### Statistical analysis

#### Descriptive statistics

The data will be analyzed descriptively and divided into SCI and control groups. Measurement or enumeration data are presented as the mean ± SEM (standard error of the mean) or number [%] (N[%]), respectively. *T tests, chi-squared tests* or *Fisher’s exact* tests were utilized for data comparisons. *P < 0.05 is regarded as a significant difference.

#### MEP and hemispheric excitability conversion degree

The MEP amplitudes of patients and controls will be analyzed by nonparametric tests. The MEP latency and central motor conduction time (CMCT) are analyzed with independent samples *t* tests. The hemispheric excitability conversion degree [ln(DH/NDH ratio)] [13] is compared with generalized estimating equations (GEEs) [27] with respect to disease course (1 m, 2 m, 4 m, 6 m, and 12 m). *P < 0.05 was regarded as a significant difference.

#### Correlation analysis and multiple linear regression analysis

Correlation analysis of hemispheric excitability conversion degree and overall functional recovery was performed to determine the effects of disease course, AIS grade, and neurological level of injury. Pearson correlations between different phases/groups were calculated after the normality test. *P < 0.05 is regarded as a significant difference.

Univariate and multivariate linear regression analyses will be conducted to validate the influence of hemispheric excitability conversion degree as an independent factor. The MBI and SCIM are set as the linear regression analysis outcomes. The inclusion criterion for univariate analysis was *P* < 0.10. *P < 0.05 is regarded as a significant difference.

### Monitoring of the safety and potential risks

TMS and fNIRS are noninvasive methods. During or after the procedure, some mild effects such as headache and dizziness may occur, which are normal reactions. If t symptoms occur, the doctor will actively treat and control them and seek assistance from relevant departments for diagnosis and treatment if necessary.

## Discussion

The present study is the first to analyze the role of bilateral M1 hand area excitability changes in the evaluation and prediction of overall functional recovery (including motor function and ADL ability) after SCI. In healthy people, the ppTMS-ICF of the DH is slightly greater than that of the NDH under physiological conditions, with no significant difference, confirming that both hemispheres generally maintain a stable status [28]. Ridding MC et al. confirmed that the DH side exhibited low-level short-interval intracortical inhibition, indicating the high excitability of DH [29]. Triggs WJ et al. also reported that the DH had a lower MEP threshold than the NDH, which suggested a high CSE in the DH [30]. In stroke, it is widely accepted that the stabilization of bilateral hemisphere excitability plays a vital role in motor rehabilitation. According to the interhemispheric inhibition model [31], the ‘overactive’ M1 of the unaffected hemisphere exhibits abnormally high interhemispheric inhibition compared with that of the affected hemisphere, impairing the recovery of the affected extremity. According to this model, one of our previous clinical study used the following protocol: the M1 hand area on the affected side with 10 Hz rTMS (excitation) and on the unaffected side with 1 Hz rTMS (inhibition). After intervention, patients showed significant improvement in motor function [32].

However, the changes in bilateral M1 excitability in individuals with SCI remain unclear, which limits targeted intervention for motor rehabilitation. To verify whether stable interhemispheric excitability in healthy people changes in individuals with SCI, we utilized spTMS (reflection of the CSE), ppTMS (reflection of intracortical excitability), and fNIRS (reflection of cortical blood flow) to assess changes in excitability and activation in the bilateral M1 hand area of individuals with SCI and its correlation with motor recovery/ADL ability.

Moreover, evaluating and predicting overall functional recovery (including motor function and ADL ability) after SCI is another target of this research. As discussed above, a recent study revealed that the M1 hand area of individuals with SCI encodes the activity information of both the UE and LE, and the movement coding of the UE and LE are highly correlated [9]. Similarly, Freund P et al. reported an increase in task-related activation in the paralyzed left M1 leg area during a right handgrip task in individuals with SCI, indicating structural associations between hand motor function and the M1 leg area [33]. In this view, the above studies support the opinion that M1 hand area and extremity motor function (including the UE and LE) are highly correlated. Therefore, changes in the hemispheric excitability of the M1 hand area may be used to assess extremity motor function and ADL ability, which could serve as potential indicators of overall functional recovery. Herein, we utilized the MS, the SCIM12 and the MAS for motor function evaluation and the MBI and SCIM for ADL ability assessment. The MS focuses on basic motor function. In addition, the total MS consists of the UEMS and LEMS, which is more convenient for distinguishing between the UE and LE. The SCIM12 evaluates the mobility of individuals with SCI indoors [25]. The MAS measures muscle tone. For ADL ability, the MBI is the most widely used scale and has been utilized to evaluate functional outcomes after SCI rehabilitation [34]. The SCIM focuses on individuals with SCI and has a relatively high sensitivity [35]. We utilize these two ADL assessment scales for mutual verification.

Based on our previous retrospective research, a correlation analysis of hemispheric excitability conversion degree and overall functional recovery was performed to investigate the effects of neurological level of injury, disease course and AIS grade. The results showed a completely different correlation tendency in patients in the SCI subgroup. Among patients with different degrees of neurological injury, patients in the noncervical injury group had a significantly positive correlation with the degree of hemispheric CSE conversion and ability to perform ADL ability, while a nonsignificant positive correlation was found in the cervical injury group. This finding may be due to the influence of cervical injury on the UE CST pathway. In patients with AIS A, the degree of hemispheric CSE conversion was significantly positively correlated with the ability to perform ADLs, while no correlation was observed in AIS non-A patients. We further observed and confirmed changes in the cortical excitability of the M1 hand area under different influencing factors over a long period of time through a prospective cohort study.

### Strengths of the study

The strengths of this study include the expansion of the theory of the predominant role of the traditional M1 area and the identification of a novel factor affecting the recovery of overall function in individuals with SCI.

First, the M1 has traditionally been thought to form a continuous somatotopic homunculus of foot-to-face representation extending along the precentral gyrus. However, as discussed above, more recent studies revealed distinct connectivity among the M1 hand area and effector-specific (foot, hand and mouth) areas [9, 10], firmly indicating the complexity of the M1 hand area. This study will increase our understanding of the relative dominance of the traditional M1 in motor control.

Second, due to the deep location and small area of the M1 LE area and the limited efficacy of the current figure-8 and circular rTMS coils [6], some studies have shown that even after rTMS stimulation of the continuous M1 LE area for 4 weeks, the gait function of individuals with SCI did not improve significantly compared with that of the sham group [4, 5]. The M1 hand area is large and shallow and is the most commonly used stimulation target in the clinic. Unfortunately, at present, only a few studies [3] have explored the improvement effect of high-frequency rTMS stimulation toward the left M1 hand area on LE movement, and the underlying mechanism is still unknown. Therefore, in contrast to current research focusing on the injured spinal cord, this study explored bilateral M1 hand area excitability changes after SCI and investigated the key mechanism of M1 hand area excitability imbalance and overall functional recovery to identify novel targets and stimulation paradigms for improving the efficacy of rTMS treatment.

### Clinical and research implications of the study

The first implication of this study will be the optimization of rTMS treatment for individuals with SCI. In stroke, it has been recommended that low-frequency rTMS of the contralesional M1 hand area (Level A) and high-frequency rTMS of the ipsilesional M1 hand area (Level B) have therapeutic effects [8]. Clarification of bilateral M1 excitability changes in individuals with SCI will provide a theoretical basis for rTMS parameter design. Moreover, this study focused on providing potential indicators (bilateral M1 excitability changes) for the early prediction of functional recovery in individuals with SCI. The early identification of bilateral M1 excitability may have predictive value for people with SCI by assisting rehabilitation clinicians in decision-making and timely and specific consultations with therapists and other health care providers.

The second aim of this study was to explore the theoretical basis of intracortical brain-computer interface design for individuals with SCI. As mentioned above, the hand knob area of the precentral gyrus (the location of the M1 area) of an individual with SCI is tuned to the entire body. Interestingly, this area contains a compositional code that links matching movements from the UE and LE. Therefore, matching movements with the M1 hand area has important implications for intracortical brain-computer interfaces (BCIs), which provides the opportunity to decode movements across the entire body from just a small area of the M1 hand region [9]. Lorach H et al. also attempted to restore communication between the brain and spinal cord with a brain–spine interface (BSI) that could induce chronic tetraplegia in individuals with SCI-related LE movement. The region that responded more strongly to the intention to move the LE identified by ECoG signals basically covered the M1 hand area [11]. Considering the studies above, clarification of bilateral M1 excitability changes in individuals with SCI can also provide a theoretical basis for intracortical BCI design.

Although promising, it is important to note that the bilateral M1 hand area excitability changes in individuals with SCI observed in this study might be disturbed by uncontrollable influencing factors due to the characteristics of observational studies; therefore, further studies targeting M1 hand area excitability modulation should also be conducted through randomized controlled trials.

### Electronic supplementary material

Below is the link to the electronic supplementary material.

Supplementary Material 1

Supplementary Material 2

Supplementary Material 3

Supplementary Material 4

Supplementary Material 5

## Data Availability

The datasets used and analyzed during the study are available from the corresponding author upon reasonable request.

## Declarations

### Current study status

Recruitment started in September 2023 and will continue throughout 2024 for the cohort. By January 17, 2024, 10 patients and 10 healthy controls had agreed to participate in the study.

## Abbreviations

SCI: Spinal Cord Injury
rTMS: repeated Transcranial Magnetic Stimulation
ADL: Activities of Daily Living
MD: Motor Dysfunction
M1: Motor Cortex
MEP: Motor Evoked Potentials
DH: Dominant Hemisphere
NDH: Non-Dominant Hemisphere
UEMS: Upper Extremity Motor Score
LEMS: Lower Extremity Motor Score
MNI: Montreal Neurological Institute
ROI: Region Of Interest
spTMS: single pulse TMS
ppTMS: paired-pulse TMS
fNIRS: functional Near-Infrared Spectroscopy
RMT: Resting Motor Threshold
MEP: Motor Evoked Potentials
AIS: American Spinal Injury Association (ASIA) impairment scale
SICI: Short-Interval Intracortical Inhibition
ICF: Intracortical Facilitation
LICI: Long-Interval Intracortical Inhibition
MAS: Modified Ashworth Scale
MBI: Modified Barthel Index
SCIM: Spinal Cord Independence Measure
CMCT: Central Motor Conduction Time
ppTMS: paired-pulse TMS
fNIRS: Functional Near-Infrared Spectroscopy
SICI: Short-Interval Intracortical Inhibition
eCRF: electronic Case Report Form
NMDA: N-methyl-D-aspartic acid
GABA_A_: Gamma-Aminobutyric Acid A
GABA_B_: Gamma-Aminobutyric Acid B

## References

[1] Singh A (2014). Global prevalence and incidence of traumatic spinal cord injury. Clin Epidemiol.

[2] Solstrand Dahlberg L (2018). Brain changes after spinal cord injury, a quantitative meta-analysis and review. Neurosci Biobehav Rev.

[3] Urbin MA (2019). What is the functional relevance of reorganization in primary motor cortex after spinal cord injury?. Neurobiol Dis.

[4] Kumru H (2016). Placebo-controlled study of rTMS combined with Lokomat((R)) gait training for treatment in subjects with motor incomplete spinal cord injury. Exp Brain Res.

[5] Krogh S (2022). Effects of repetitive transcranial magnetic stimulation on recovery in lower limb muscle strength and gait function following spinal cord injury: a randomized controlled trial. Spinal Cord.

[6] Rossi S (2021). Safety and recommendations for TMS use in healthy subjects and patient populations, with updates on training, ethical and regulatory issues: Expert guidelines. Clin Neurophysiol.

[7] Roth Y (2007). Three-dimensional distribution of the electric field induced in the brain by transcranial magnetic stimulation using figure-8 and deep H-coils. J Clin Neurophysiol.

[8] Lefaucheur JP (2020). Evidence-based guidelines on the therapeutic use of repetitive transcranial magnetic stimulation (rTMS): an update (2014–2018). Clin Neurophysiol.

[9] Willett FR (2020). Hand Knob Area of Premotor Cortex represents the whole body in a compositional way. Cell.

[10] Gordon EM (2023). A somato-cognitive action network alternates with effector regions in motor cortex. Nature.

[11] Lorach H (2023). Walking naturally after spinal cord injury using a brain-spine interface. Nature.

[12] PENFIELD W, BOLDREY E (1937). Somatic motor and sensory representation in the cerebral cortex of man as studied by electrical stimulation. Brain.

[13] Dai CQ (2023). Primary motor hand area corticospinal excitability indicates overall functional recovery after spinal cord injury. Front Neurol.

[14] Chan AW (2013). SPIRIT 2013 explanation and elaboration: guidance for protocols of clinical trials. BMJ.

[15] Zalewski NL (2019). Characteristics of spontaneous spinal cord infarction and proposed diagnostic criteria. JAMA Neurol.

[16] Oyama K, Hu L, Sakatani K (2018). Prediction of MMSE score using time-resolved Near-Infrared Spectroscopy. Adv Exp Med Biol.

[17] Ziemann U (2015). TMS and drugs revisited 2014. Clin Neurophysiol.

[18] Martinez-Calderon J (2017). Influence of psychological factors on the prognosis of chronic shoulder pain: protocol for a prospective cohort study. BMJ Open.

[19] Sun X (2019). Analgesia-enhancing effects of repetitive transcranial magnetic stimulation on neuropathic pain after spinal cord injury:an fNIRS study. Restor Neurol Neurosci.

[20] Premoli I (2018). Short-interval and long-interval intracortical inhibition of TMS-evoked EEG potentials. Brain Stimul.

[21] Drew PJ (2022). Neurovascular coupling: motive unknown. Trends Neurosci.

[22] Meseguer-Henarejos AB (2018). Inter- and intra-rater reliability of the Modified Ashworth Scale: a systematic review and meta-analysis. Eur J Phys Rehabil Med.

[23] Roberts TT, Leonard GR, Cepela DJ (2017). Classifications in brief: American Spinal Injury Association (ASIA) impairment scale. Clin Orthop Relat Res.

[24] Kapadia N (2014). A randomized trial of functional electrical stimulation for walking in incomplete spinal cord injury: effects on walking competency. J Spinal Cord Med.

[25] van Middendorp JJ (2011). A clinical prediction rule for ambulation outcomes after traumatic spinal cord injury: a longitudinal cohort study. Lancet.

[26] Prodinger B (2017). Establishing score equivalence of the functional independence measure motor scale and the Barthel Index, utilising the International Classification of Functioning, disability and health and Rasch measurement theory. J Rehabil Med.

[27] Ito T, Sugasawa S. Grouped generalized estimating equations for longitudinal data analysis. Biometrics, 2022. 10.1111/biom.13718 35819419

[28] Civardi C (2000). Hemispheric asymmetries of cortico-cortical connections in human hand motor areas. Clin Neurophysiol.

[29] Ridding MC, Flavel SC (2006). Induction of plasticity in the dominant and non-dominant motor cortices of humans. Exp Brain Res.

[30] Triggs WJ, Calvanio R, Levine M (1997). Transcranial magnetic stimulation reveals a hemispheric asymmetry correlate of intermanual differences in motor performance. Neuropsychologia.

[31] Nowak DA (2010). Noninvasive brain stimulation and motor recovery after stroke. Restor Neurol Neurosci.

[32] Long H (2018). Effects of combining high- and low-frequency repetitive transcranial magnetic stimulation on upper limb hemiparesis in the early phase of stroke. Restor Neurol Neurosci.

[33] Freund P (2012). Axonal integrity predicts cortical reorganisation following cervical injury. J Neurol Neurosurg Psychiatry.

[34] Anderson K (2008). Functional recovery measures for spinal cord injury: an evidence-based review for clinical practice and research. J Spinal Cord Med.

[35] Unai K (2019). Association between SCIM III Total Scores and Individual Item Scores to predict independence with ADLs in persons with spinal cord Injury. Arch Rehabil Res Clin Transl.

