# Supplementary material for: Correlation of bilateral M1 hand area excitability and overall functional recovery after spinal cord injury: protocol for a prospective cohort study
Source: BMC Neurol. 2024 Jun 22;24:213. doi: 10.1186/s12883-024-03705-0 (PMC11193300; doi:10.1186/s12883-024-03705-0)
Supplement: Supplementary file 1 — Supplementary Material 1 [file 12883_2024_3705_MOESM1_ESM.docx]

**Motor Score Evaluation of Xijing Hospital**

**Name_ _ _ Gender_ _ _ Age_ _ _ Department_ _ _ Bed No. _ _ _**

**Patient No. _ _ _clinical diagnosis_ _ _ _**

**Date:**

| **Evaluation muscle** | **Left** | **Right** | **Evaluation muscle** | **Left** | **Right** |
| --- | --- | --- | --- | --- | --- |
| elbow flexor |  |  | hip flexor |  |  |
| wrist extensor |  |  | knee extensor |  |  |
| elbow extensor |  |  | ankle back extensor |  |  |
| middle finger flexor |  |  | extensor pollicis longus |  |  |
| little finger abductor |  |  | ankle plantar flexor |  |  |
