# Supplementary material for: Correlation of bilateral M1 hand area excitability and overall functional recovery after spinal cord injury: protocol for a prospective cohort study
Source: BMC Neurol. 2024 Jun 22;24:213. doi: 10.1186/s12883-024-03705-0 (PMC11193300; doi:10.1186/s12883-024-03705-0)
Supplement: Supplementary file 2 — Supplementary Material 2 [file 12883_2024_3705_MOESM2_ESM.docx]

**Modified Ashworth Scale Evaluation of Xijing Hospital**

**Name_ _ _ Gender_ _ _ Age_ _ _ Department_ _ _ Bed No. _ _ _**

**Patient No. _ _ _clinical diagnosis_ _ _ _**

**Date:**

| **Evaluated limb** | **Scale** |
| --- | --- |
| Left upper extremity |  |
| Right upper extremity |  |
| Left lower extremity |  |
| Right lower extremity |  |

Modified Ashworth scale evaluation criteria

| Scale | Criteria |
| --- | --- |
| 0 | muscle tension is completely normal |
| 1 | muscle tension is slightly higher than normal people, the minimum resistance can be felt within its range of motion when flexing the joint passively |
| 1+ | slight increase in muscle tension, sudden seizure can be felt within 50% of the range of joint activity, or minimum resistance at 50% of the range of joint activity can be felt |
| 2 | the muscle tension increases obviously. In most motion range of the joint, the muscle tension increase, but the affected part can still carry out passive movement easily |
| 3 | muscle tension increases significantly and passive activity was difficult |
| 4 | the joint is stiff, completely unable to move, and the affected part shows obvious stiffness during passive flexion |
