# Supplementary material for: Correlation of bilateral M1 hand area excitability and overall functional recovery after spinal cord injury: protocol for a prospective cohort study
Source: BMC Neurol. 2024 Jun 22;24:213. doi: 10.1186/s12883-024-03705-0 (PMC11193300; doi:10.1186/s12883-024-03705-0)
Supplement: Supplementary file 3 — Supplementary Material 3 [file 12883_2024_3705_MOESM3_ESM.docx]

**Spinal Cord Injury (SCI) Functional Assessment of Xijing Hospital**

**Name_ _ _ Gender_ _ _ Age_ _ _ Department_ _ _ Bed No. _ _ _**

**Patient No. _ _ _clinical diagnosis_ _ _ _**

**Date:**

| **L R**   \|  \| C5 \|  \| elbow flexor \| \| --- \| --- \| --- \| --- \| \|  \| C6 \|  \| wrist extensor \| \|  \| C7 \|  \| elbow extensor \| \|  \| C8 \|  \| middle finger flexor \| \|  \| T1 \|  \| little finger abductor \| \|  \|  \|  \|  \| \|  \| L2 \|  \| hip flexor \| \|  \| L3 \|  \| knee extensor \| \|  \| L4 \|  \| ankle back extensor \| \|  \| L5 \|  \| extensor pollicis longus \| \|  \| S1 \|  \| ankle plantar flexor \|   Sacral movement：yes □ no □   \|  \| + \|  \| = \|  \| **Motor Score** \| \| --- \| --- \| --- \| --- \| --- \| --- \|   **(50) (50) (100)** | **1.neurological level：**   \|  \| left \| right \| \| --- \| --- \| --- \| \| sensory \|  \|  \| \| motor \|  \|  \|   **2. AIS grade：**  **3.Rehabilitation issues：** | **Light touch Pin Prick**  L R L R   \|  \|  \|  \| C2 \|  \|  \|  \| \| --- \| --- \| --- \| --- \| --- \| --- \| --- \| \|  \|  \|  \| C3 \|  \|  \|  \| \|  \|  \|  \| C4 \|  \|  \|  \| \|  \|  \|  \| C5 \|  \|  \|  \| \|  \|  \|  \| C6 \|  \|  \|  \| \|  \|  \|  \| C7 \|  \|  \|  \| \|  \|  \|  \| C8 \|  \|  \|  \| \|  \|  \|  \| T1 \|  \|  \|  \| \|  \|  \|  \| T2 \|  \|  \|  \| \|  \|  \|  \| T3 \|  \|  \|  \| \|  \|  \|  \| T4 \|  \|  \|  \| \|  \|  \|  \| T5 \|  \|  \|  \| \|  \|  \|  \| T6 \|  \|  \|  \| \|  \|  \|  \| T7 \|  \|  \|  \| \|  \|  \|  \| T8 \|  \|  \|  \| \|  \|  \|  \| T9 \|  \|  \|  \| \|  \|  \|  \| T10 \|  \|  \|  \| \|  \|  \|  \| T11 \|  \|  \|  \| \|  \|  \|  \| T12 \|  \|  \|  \| \|  \|  \|  \| L1 \|  \|  \|  \| \|  \|  \|  \| L2 \|  \|  \|  \| \|  \|  \|  \| L3 \|  \|  \|  \| \|  \|  \|  \| L4 \|  \|  \|  \| \|  \|  \|  \| L5 \|  \|  \|  \| \|  \|  \|  \| S1 \|  \|  \|  \| \|  \|  \|  \| S2 \|  \|  \|  \| \|  \|  \|  \| S3 \|  \|  \|  \| \|  \|  \|  \| S4-5 \|  \|  \|  \| | **Muscle tone**  L R   \|  \|  \|  \| Ashworth \| \| --- \| --- \| --- \| --- \| \| **Clonus**  L R \| \| \| \| \|  \|  \|  \| Patellar clonus \| \|  \|  \|  \| Ankle clonus \|   **Reflex**  L R   \|  \|  \|  \| Biceps reflex \| \| --- \| --- \| --- \| --- \| \|  \|  \|  \| Triceps reflex \| \|  \|  \|  \| Radial periosteal reflex \| \|  \|  \|  \| Abdominal reflex \| \|  \|  \|  \| Cremasteric reflex \| \|  \|  \|  \| Knee reflex \| \|  \|  \|  \| Ankle reflex \| \|  \|  \|  \| Hoffman \| \|  \|  \|  \| Babinski \| \|  \| \| \| \| \| M \|  \|  \| Bulbar anal reflex \| \| F \|  \|  \| Clitoral anal reflex \| \| Partial reserved area \| \| \| \|   L：  R：  Sacral sensation：Yes□ No□ |
| --- | --- | --- | --- | --- | --- | --- | --- | --- | --- | --- | --- | --- | --- | --- | --- | --- | --- | --- | --- | --- | --- | --- | --- | --- | --- | --- | --- | --- | --- | --- | --- | --- | --- | --- | --- | --- | --- | --- | --- | --- | --- | --- | --- | --- | --- | --- | --- | --- | --- | --- | --- | --- | --- | --- | --- | --- | --- | --- | --- | --- | --- | --- | --- | --- | --- | --- | --- | --- | --- | --- | --- | --- | --- | --- | --- | --- | --- | --- | --- | --- | --- | --- | --- | --- | --- | --- | --- | --- | --- | --- | --- | --- | --- | --- | --- | --- | --- | --- | --- | --- | --- | --- | --- | --- | --- | --- | --- | --- | --- | --- | --- | --- | --- | --- | --- | --- | --- | --- | --- | --- | --- | --- | --- | --- | --- | --- | --- | --- | --- | --- | --- | --- | --- | --- | --- | --- | --- | --- | --- | --- | --- | --- | --- | --- | --- | --- | --- | --- | --- | --- | --- | --- | --- | --- | --- | --- | --- | --- | --- | --- | --- | --- | --- | --- | --- | --- | --- | --- | --- | --- | --- | --- | --- | --- | --- | --- | --- | --- | --- | --- | --- | --- | --- | --- | --- | --- | --- | --- | --- | --- | --- | --- | --- | --- | --- | --- | --- | --- | --- | --- | --- | --- | --- | --- | --- | --- | --- | --- | --- | --- | --- | --- | --- | --- | --- | --- | --- | --- | --- | --- | --- | --- | --- | --- | --- | --- | --- | --- | --- | --- | --- | --- | --- | --- | --- | --- | --- | --- | --- | --- | --- | --- | --- | --- | --- | --- | --- | --- | --- | --- | --- | --- | --- | --- | --- | --- | --- | --- | --- | --- | --- | --- | --- | --- | --- | --- | --- | --- | --- | --- | --- | --- | --- | --- | --- | --- | --- | --- | --- | --- | --- | --- | --- | --- | --- | --- | --- | --- | --- | --- | --- | --- | --- | --- | --- | --- | --- | --- | --- | --- | --- | --- | --- | --- | --- | --- | --- | --- | --- | --- | --- | --- | --- | --- | --- | --- | --- | --- | --- | --- | --- | --- | --- | --- | --- | --- |
| Bladder：  Intestine：  Pressure ulcers：  Pain：  Thermoregulation disorders:  ：  Cardiovascular regulatory disorders:  ：  Infection (lung or Urinary system):  ：  ROM (normal/abnormal)： | |  |  |
|  |  |  |  |
|  |  | \|  \| + \|  \|  \| = \|  \| **Light touch total** \| \| --- \| --- \| --- \| --- \| --- \| --- \| --- \|   **(56) (56) (112)**   \|  \|  \|  \| + \|  \| = \|  \| **Pin Prick total** \| \| --- \| --- \| --- \| --- \| --- \| --- \| --- \| --- \|   **(56) (56) (112)** | |
