# Supplementary material for: Correlation of bilateral M1 hand area excitability and overall functional recovery after spinal cord injury: protocol for a prospective cohort study
Source: BMC Neurol. 2024 Jun 22;24:213. doi: 10.1186/s12883-024-03705-0 (PMC11193300; doi:10.1186/s12883-024-03705-0)
Supplement: Supplementary file 4 — Supplementary Material 4 [file 12883_2024_3705_MOESM4_ESM.docx]

**Spinal Cord Injury Independence Measurement (SCIM)**

**of Xijing Hospital**

**Name_ _ _ Gender_ _ _ Age_ _ _ Department_ _ _ Bed No. _ _ _**

**Patient No. _ _ _clinical diagnosis_ _ _ _**

**Date:**

| **Task** | **Scores** | **Notes** |
| --- | --- | --- |
| Feeding |  |  |
| Bathing |  |  |
| Dressing |  |  |
| Grooming |  |  |
| Respiration |  |  |
| Sphincter Management-Bladder |  |  |
| Sphincter Management-bowel |  |  |
| Use of toilet |  |  |
| Motion in bed and sore prevention |  |  |
| Transfers: bed-wheelchair |  |  |
| Transfers: wheelchair-toilet-tub |  |  |
| Mobility indoors |  |  |
| Mobility for moderate distances |  |  |
| Mobility outdoors |  |  |
| Stair management |  |  |
| Transfers: wheelchair-car |  |  |
| Transfers: ground-wheelchair |  |  |
| Total |  |  |
