# Supplementary material for: Correlation of bilateral M1 hand area excitability and overall functional recovery after spinal cord injury: protocol for a prospective cohort study
Source: BMC Neurol. 2024 Jun 22;24:213. doi: 10.1186/s12883-024-03705-0 (PMC11193300; doi:10.1186/s12883-024-03705-0)
Supplement: Supplementary file 5 — Supplementary Material 5 [file 12883_2024_3705_MOESM5_ESM.docx]

**Modified Barthel Index (MBI) Assessment of Xijing Hospital**

**Name_ _ _ Gender_ _ _ Age_ _ _ Department_ _ _ Bed No. _ _ _**

**Patient No. _ _ _clinical diagnosis_ _ _ _**

**Date:**

| **Task** | **Scoring criteria** | **scores** |
| --- | --- | --- |
| 1 Feeding | Fully independent 10, Minor assistance 8, Moderate help 5, A lot of help 2, Fully dependent on 0 |  |
| 2 Bathing | Fully independent 5, Minor assistance 4, Moderate help 3, A lot of help 1, Fully dependent on 0 |  |
| 3 Personal hygiene | Fully independent 5, Minor assistance 4, Moderate help 3, A lot of help 1, Fully dependent on 0 |  |
| 4 Dressing | Fully independent 10, Minor assistance 8, Moderate help 5, A lot of help 2, Fully dependent on 0 |  |
| 5 Bowel control | Fully independent 10, Minor assistance 8, Moderate help 5, A lot of help 2, Fully dependent on 0 |  |
| 6 Bladder control | Fully independent 10, Minor assistance 8, Moderate help 5, A lot of help 2, Fully dependent on 0 |  |
| 7 Toilet | Fully independent 10, Minor assistance 8, Moderate help 5, A lot of help 2, Fully dependent on 0 |  |
| 8 Transfer ability | Fully independent 15, Minor assistance 12, Moderate help 8, A lot of help 3, Fully dependent on 0 |  |
| 9 Mobility for distances | Fully independent 15, Minor assistance 12, Moderate help 8, A lot of help 3, Fully dependent on 0 |  |
| 9* Wheelchair control | Fully independent 5, Minor assistance 4, Moderate help 3, A lot of help 1, Fully dependent on 0 |  |
| 10 Stairs climbing | Fully independent 10, Minor assistance 8, Moderate help 5, A lot of help 2, Fully dependent on 0 |  |
| Total |  |  |
